# Editorial: Sexual dimorphism of the immune inflammatory response in infectious and non-infectious diseases, volume II

**DOI:** 10.3389/fimmu.2023.1341365

**Published:** 2023-11-30

**Authors:** Mustapha Chamekh, Georges Casimir

**Affiliations:** ^1^ Inflammation Unit, Laboratory of Pediatric Research and ULB Center for Research in Immunology (U-CRI), Faculty of Medicine, Brussels, Belgium; ^2^ Laboratory of Pediatric Research, Faculty of Medicine, University Children’s Hospital HUDERF, Université Libre de Bruxelles (ULB), Brussels, Belgium

**Keywords:** inflammatory disease, infections disease, immune response, chromosome X-linked genes, sexual hormone

Accumulating evidence unequivocally indicates that male and female people are not equal when it comes to many infectious and non-infectious inflammatory diseases. The recent example of the COVID-19 pandemic has shed light on this discrepancy and brought this concept back to center stage, showing how the disease can take wildly different paths in male and female people. It is no surprise, then, that sex disparities in pathological manifestations have sparked growing interest over the last years. When considering sex as a biological variable in study design, many clinical and preclinical studies reported that the majority of fatalities in acute and chronic inflammatory diseases occurred among male and female people, respectively. This is mainly attributed to sex differences in the type and magnitude of the innate and adaptive host immune response triggered during infection or trauma. It is widely agreed that the immune inflammatory response is a double-edged sword in that it is crucial to fight the infection and repair the injury, while it might be deleterious when excessive and not tightly controlled. Noteworthily, sex disparities in inflammatory diseases have been reported across the life span, including the prepubescent period, hence supporting the prominent role of the chromosome X-linked genetic architecture that may act synergistically with sex hormones later in life. In support of this, chromosome X harbors genes playing a key role in innate and adaptive immunity, such as IRAK-1, Foxp3, and CD40 ligand. Chromosome X is also rich in gene sequences coding for microRNAs (miRNA) that are known to play a crucial role in the post-transcriptional regulation of several genes involved in various biological processes, including inflammation. Despite the dosage compensation, which takes place in female people thanks to the inactivation of one of the X chromosomes, the fact remains that certain genes can escape the inactivation process. Consequently, the overexpression of certain X-linked genes in individuals with two copies of the X chromosome but not in those with a single X chromosome could have an impact on the triggering and modulation of the immune response.

The articles presented in volume II of the Research Topic complete the series of previously published reviews in volume I (1). They cover different facets of the sexual dimorphism of the inflammatory response in various diseases and highlight immune-related pathogenic mechanisms. In viral infections, type I interferon responses mediated by innate immune cells play a cardinal role in host defense. The article by Pujantell and Altfeld addresses the contribution of differences in the type I IFN pathway between male and female people to the regulation of antiviral immunity and its implication for sex-related outcomes. The authors place a particular focus on the effects of sex hormones and genes encoded by the X chromosome. Beyond describing the occurrence of sex-related differential outcomes in inflammatory diseases in early life, the report by Kelly et al. sets out to illustrate how being male is a risk factor for neonatal brain injury and associated inflammation. The authors outline the gaps in clinical and laboratory studies in considering sex as a biological variable for the analysis of brain injury outcomes and related immune responses. Furthermore, they describe current immunomodulatory therapies, some of which show sex-specific responses for preterm and term infants, making them a promising strategy for optimizing treatments.

On the other hand, different reviews and original reports presented here on chronic inflammatory diseases illustrate how female people exhibit more vulnerability compared to male people. Young et al. address the example of Hidradenitis suppurativa (HS), a chronic inflammatory disease that affects the skin. The hallmark of the disease is an immune dysregulation with elevated pro-inflammatory cytokines associated with T helper (Th) cells, especially Th1- and Th17-mediated immunity. The authors discuss the potential immunological mechanisms in HS pathophysiology by addressing the effects of hormones, X chromosome dosage, genetics, the microbiome, and smoking on sex-related differences in immunity. Likewise, the disease severity of allergic asthma is more prevalent in female people compared to male people. The investigation by Hemshekhar et al. provides important insights into sex-related differences in allergen-mediated protein biomarkers within the lungs in animal and human studies. Sex disparity has been also reported in cystic fibrosis (CF) disease, with female people experiencing frequent pulmonary exacerbations associated with microbial infections, resulting in shorter survival expectancy. A pilot study by Deny et al. investigates whether chromosome X-linked miRNAs could be linked to sex-related differential outcomes. They report sex-biased expression of miRNAs with a potent regulatory effect on the inflammatory response. Given the important role of modifier genes in disease progression, studying the miRNA profile according to sex will pave the path to refining disease stratification.

Including sex as a biological variable in bio-clinical investigations provides critical insights into a patient’s risk of disease relapse or progression and, therefore, should allow the optimization of clinical monitoring. Thus, considering the stratification of data and healthcare statistics by sex will fill gaps in knowledge of the impact of sex on incidence and outcomes and will certainly help to not gloss over inequities and their causes.

## Author contributions

MC: Writing – review & editing. GC: Writing – review & editing.
